# The Chernobyl Accident 20 Years On: An Assessment of the Health Consequences and the International Response

**DOI:** 10.1289/ehp.9113

**Published:** 2006-05-30

**Authors:** Keith Baverstock, Dillwyn Williams

**Affiliations:** 1 Department of Environmental Sciences, Faculty of Natural and Environmental Sciences, University of Kuopio, Kuopio, Finland; 2 Strangeways Research Laboratory, Worts Causeway, Cambridge, United Kingdom

**Keywords:** Chernobyl, disaster response, nuclear accidents, radiation, thyroid cancer, United Nations

## Abstract

**Background:**

The Chernobyl accident in 1986 caused widespread radioactive contamination and enormous concern. Twenty years later, the World Health Organization and the International Atomic Energy Authority issued a generally reassuring statement about the consequences. Accurate assessment of the consequences is important to the current debate on nuclear power.

**Objectives:**

Our objectives in this study were to evaluate the health impact of the Chernobyl accident, assess the international response to the accident, and consider how to improve responses to future accidents.

**Discussion:**

So far, radiation to the thyroid from radioisotopes of iodine has caused several thousand cases of thyroid cancer but very few deaths; exposed children were most susceptible. The focus on thyroid cancer has diverted attention from possible nonthyroid effects, such as mini-satellite instability, which is potentially important. The international response to the accident was inadequate and uncoordinated, and has been unjustifiably reassuring. Accurate assessment of Chernobyl’s future health effects is not currently possible in the light of dose uncertainties, current debates over radiation actions, and the lessons from the late consequences of atomic bomb exposure.

**Conclusions:**

Because of the uncertainties over the dose from and the consequences of the Chernobyl accident, it is essential that investigations of its effects should be broadened and supported for the long term. Because of the problems with the international response to Chernobyl, the United Nations should initiate an independent review of the actions and assignments of the agencies concerned, with recommendations for dealing with future international-scale accidents. These should involve independent scientists and ensure cooperation rather than rivalry.

April 26, 2006, was the 20th anniversary of the Chernobyl accident, the second major single exposure to radiation of a substantial population. It is relevant to the current view of the consequences of Chernobyl to reflect on the understanding in 1965 of the health consequences of the first major event, radiation from the atomic bombs in Hiroshima and Nagasaki, Japan, in 1945. The only significant consequences observed in survivors 20 years after the atomic bombs were increases in leukemia and thyroid cancer, and the general view of the future was reassuring. In 1974, a significant increase in solid cancers was detected, and nearly 50 years after the event, an unexpected increase was found in noncancer diseases (Shimizu et al. 1992). Today, leukemia and thyroid cancer form only a small fraction of the accepted total radiation-related health detriment.

In 1990, four years after the Chernobyl accident, an increase in thyroid cancer was found in children exposed to fallout from the accident [International Atomic Energy Authority (IAEA) 1991]. Two years later, the first reports in the Western literature of an increase in childhood thyroid cancer (CTC) in Belarus were published (Baverstock et al. 1992; Kazakov et al. 1992). In 2000, about 2,000 cases of thyroid cancer had been reported in those exposed as children in the former Soviet Socialist Union, and in 2005, the number was estimated at 4,000 [World Health Organization (WHO) 2005a]; the latest estimate for the year 2056 ranges from 3,400 to 72,000 (Cardis et al. 2006). The effects are not limited by national borders; Poland has recorded cases (Niedziela et al. 2004) in spite of a rapid precautionary distribution of stable iodine (Nauman and Wolff 1993). The causative agent, ^131^I, was detected in many European countries with as yet unknown effects. Interestingly, a significant increase in leukemia has not been reliably reported in the three most affected countries.

This dramatic contrast between the two incidents is in part due to the different types of radiation exposure, but both show that the effects of massive exposures to radiation are immensely complex. In comparing the health effects after Chernobyl with those after the atomic bombs, it must be remembered that apart from workers in or close to the power plant, the Chernobyl accident involved mainly exposure to radioactive isotopes, and the atomic bombs primarily involved direct exposure to γ-rays and neutrons. Because of the prominence given to thyroid carcinoma after Chernobyl, less attention has been given to whole-body exposure from the ingestion and inhalation of all isotopes, together with the shine from the radioactive cloud and deposited radioactivity. Consideration of the health effects of Chernobyl must take into account both tissue-specific doses due to isotope concentration and whole-body doses. The most prominent tissue-specific dose is that to the thyroid, largely from ^131^I, with a smaller contribution from short-lived isotopes of iodine. For many in the 30-km zone (135,000), there were relatively high absorbed doses to other organs as well as the thyroid until evacuation (Baverstock and Williams 2003), and for those living in the contaminated areas around the 30-km zone (5 million), relatively high dose rate exposure (days to weeks) was followed by prolonged (years) exposure to a low dose rate. This exposure was a complex mixture of external radiation and internal emitters. For others living farther from the accident, in Western Europe, for example, their average exposure was equivalent to an additional ≤50% of average annual natural background level of radiation. About 600,000 liquidators assisted with the cleanup. Those working at the site shortly after the accident (200,000) received substantial doses. For all of these groups, estimates of numbers of fatal cancers can be derived from the collective doses. However, such estimates depend on the assumed risk coefficient, but of the order of 60,000 such fatalities in total can be estimated, based on the collective dose estimated by the United Nations Scientific Committee on the Effects of Atomic Radiation (UNSCEAR 1988), less than half of which would derive from the declared contaminated areas. A more recent estimate of the numbers of fatal cancers based on a collective dose of less than half the UNSCEAR estimate gives a central value of 16,000 (95% confidence interval, 7,000–38,000) (Cardis et al. 2006).

In this commentary, we will assess the established health consequences of the accident; identify some of the unanswered health issues; assess whether there are effects yet to be realized; evaluate the international response; and consider how to improve the response to future accidents.

## Health Consequences

### Firmly established

#### Thyroid carcinoma

By far, the most prominent health consequence of the accident is the increase in thyroid cancer among those exposed as children. The medical authorities in Belarus and Ukraine were aware in 1990 that the incidence of the rare (typically about 1/10^6^children/year) CTC was increasing, particularly in children living close to the reactor (IAEA 1991). Initially, various non–Chernobyl-related causes were suggested for the increase in thyroid carcinoma. In terms of radiation dose, the most likely culprit was ^131^I, a copious product of nuclear fission with an 8.1-day half-life. ^131^I is rapidly taken up by the thyroid but was widely regarded as carrying little risk of thyroid neoplasia. Swedish epidemiologic studies of the widespread use of ^131^I in diagnosis and treatment of thyroid disease found no significant risk of thyroid cancer (Holm 1999). Other isotopes of iodine and tellurium-132 were also released in very large amounts, but because of their much shorter half-lives, their most significant contribution to the thyroid dose occurred only in those living near the reactor.

The first reports of the increase in Belarus (Baverstock et al. 1992; Kazakov et al. 1992) were received with skepticism by the scientific community, but the risks were shown to be real (Williams 1996). Analysis of thyroid carcinogenesis after X-ray exposure also showed clearly that the younger the subject at exposure, the higher the risk (Ron et al. 1995). The almost complete lack of children in the Swedish studies thus accounted for the apparent lack of a carcinogenic risk from ^131^I. It has since become increasingly clear that ^131^I is as carcinogenic in children as X rays (Cardis et al. 2005a). The child’s thyroid is one of the most sensitive human tissues to cancer induction by radiation. Because iodine is a volatile element, its release from fractured fuel rods is inevitable.

Much has been made of the fact that differentiated thyroid cancer is an eminently curable disease. Only a very small number of deaths from Chernobyl-related thyroid carcinoma have occurred so far. However, the preferred treatment regime, total thyroidectomy followed by ^131^I treatment to destroy metastases, is not always fully effective. Death from papillary carcinoma of the thyroid is rare, usually of the order of 5–10%. Because of the slow growth of the tumor, it is premature to assume that the even lower death rate for current Chernobyl-related cases will be maintained, particularly for cases yet to occur. An older age at onset can be associated with a less favorable prognosis. Currently, those exposed as small children are now adolescents or young adults but continue to carry an increased risk of developing thyroid carcinoma. The incidence of thyroid cancer in those who were adults at the time of exposure is reported to have increased in the many exposed populations (Mahoney et al. 2004), although the relationship to radiation is not clear. Screening has become more sophisticated, and increased ascertainment may be a major factor (Jacob et al. 2006). The concentration of effort on the major increase in those exposed as children has meant that the possible much smaller risk to adults has not been adequately investigated.

#### Acute radiation sickness

A small group of liquidators and plant workers received very high whole-body doses. Among these, about 150 individuals were treated for acute radiation sickness; 28 of these died within a relatively short time (WHO 2005a). Approximately 20 more have since died from probable radiation-related diseases.

#### Psychological consequences

Psychological effects are of considerable importance (Havenaar et al. 1997). They arise from an understandable fear of exposure to an unknown amount of an intangible but potentially dangerous agent, fear for exposed children, mistrust of reassurances from the authorities, and for hundreds of thousands of people, the consequences of forced evacuation from home and land. For some, the stress from these experiences has precipitated psychological illness; for others, an increased consumption of alcohol and cigarettes; and for still others, dietary changes to avoid perceived contamination. Some deaths from suicide, cirrhosis, or lung cancer could be regarded as indirect consequences of the accident and the subsequent measures taken. Whatever the view the nuclear industry may have about the irrationality of these consequences, they are real and have an important impact on public health, and so deserve greater attention.

#### Genetic consequences

Another consequence, not as firmly established as thyroid cancer, is mini-satellite instability (MSI) in children born to exposed fathers after Chernobyl (Dubrova et al. 1996, 1997, 2002b). MSI is not a classical genetic effect, and its implications for health are far from clear. A similar effect has been seen in the children and grandchildren of men exposed to weapons testing in Semipalatinsk (Dubrova et al. 2002a) and a parallel phenomenon, tandem repeat instability, occurs in laboratory mice (Barber et al. 2002). MSI has not been observed in the survivors of the atomic bombings (Kodaira et al. 1995), in studies of Chernobyl cleanup workers (Livshits et al. 2001), or in radiotherapy patients (May et al. 2000). MSI is considerably more frequent in relation to radiation dose than classical genetic effects and apparently does not become diluted in subsequent generations. Although its clinical significance is uncertain (Bouffler et al. 2006), it is of some concern, certainly more than the Chernobyl Forum (WHO 2005a) gave it credit for.

These issues are particularly relevant in view of developments in radiobiological research over the past 15 years. The apparently simple relationship between radiation dose and its effects are being reappraised. In the early 1990s, two previously unacknowledged effects of radiation were reported, genomic instability and the bystander effect (Appendix). These effects are not accommodated by the current theoretical framework. Also in 1986, the risk per unit dose accrued from Chernobyl would have been assumed to be half that estimated from the atomic bombs in Japan. A recent detailed analysis of the Japanese experience suggested that the risk for those exposed to the lower doses (Pierce and Preston 2000) could even be supralinear (Brenner et al. 2003; Brenner and Sachs 2006). Furthermore, the accuracy of the standard models for inferring doses from internal exposure have been questioned by the U.K. Committee Examining Radiation Risk of Internal Emitters (CERRIE 2004). There is, therefore, considerable uncertainty in translating collective dose to health detriment and fatalities.

### Unanswered issues

#### Birth defects

There have been many claims of an increased incidence of congenital anomalies in children born shortly after the accident. Some cases reported in the press show abnormalities similar to those following the use of thalidomide in pregnancy, and thalidomide was apparently available in the Soviet Union. It is not possible to separate Chernobyl-related abnormalities from those due to other causes or from the effects of increased ascertainment. Although a slight increase in minor conditions has been observed, there does not appear to have been a major increase in serious conditions such as limb deformities.

#### Leukemia

Intensive efforts have been made to detect an increase in leukemia, which is strongly associated with radiation. No statistically significant increases of those forms associated with radiation have been reported, but increases in chronic lymphatic leukemia, a non–radiation-related disease of older age, may testify to increased case ascertainment (WHO 2005a). However, the level of increase expected, given the received doses, anticipated risk factor, and the rarity of the condition, would only be detected by large analytical—as opposed to ecological—epidemiology studies.

### In the future

Experience from Japan shows that many effects of whole-body radiation exposure may not be apparent for decades. While the short initial latent period associated with the thyroid carcinoma after Chernobyl, together with the very large amounts of radioactive isotopes of iodine released, have led to a huge effort to reconstruct thyroid doses, much less attention has been paid to whole-body doses. Measurements of the initial exposure phase for those in the 30-km zone, while confused, point to absorbed doses to the whole body of many individuals that were > 1 Gy, with average doses to some 25,000 Belarusian evacuees of a substantial fraction of a Gray (Baverstock and Williams 2003). Doses received by infants evacuated from the 30-km zone are estimated to be in the range of 0.03–2 Sv (Mück et al. 2002; Pröhl et al. 2002), well within the range that led to a significant rise in cancer incidence after the atomic bombs. As well as the thyroid, other organs show some concentration of iodine. One particularly important tissue is breast epithelium, which can concentrate iodine and receive γ-radiation from isotopes in the lung or thyroid. Some particular groups at exposure may show an excess incidence of breast cancer now or in the future (Pukkala et al. 2006). A significant rise in incidence of a range of malignancies in the population exposed to high levels of fallout, particularly those exposed as children, is clearly possible. All too often the phrase “no increase has been observed” conceals the lack of an adequate study.

The full complexity of the exposure regime has not been adequately explored, and the estimation of whole-body and many tissue-specific doses is imprecise or unknown. The radiation dose received from the atomic bombs was still being revised 50 years after the event. Taking into account the results of new research and the CERRIE report (CERRIE 2004), it is very difficult to derive with any confidence the likely levels of health detriment from the estimated dose levels. It is also too soon to make an accurate assessment of longer-term effects from those already observed.

In the light of this level of uncertainty, the case is compelling for international research surveillance of the millions of people exposed to fallout from Chernobyl and selective follow-up of those exposed to high levels similar to that following the atomic bombings in Japan (Baverstock 1998; Williams 2002; Williams and Baverstock 2006).

## The International Response

An accident on the scale of Chernobyl would be a challenge to most countries. However, the Union of Soviet Socialist Republics (USSR) felt able to deal with the consequences, at least up until 1989, when it sought assistance from the WHO and the IAEA to evaluate the consequences of the accident in environmental and health terms. In response, the IAEA created the International Chernobyl Project, which oversaw a visit to the affected areas and made a comprehensive report on radiological consequences and protective measures (IAEA 1991). The team seems then to have been disbanded. Public concern was widespread, and the questions posed by the public to IAEA expert panels at public meetings show the extent of this concern (IAEA 1991). Following the breakup of the USSR, the consequences became the responsibility of three newly independent states: Ukraine, Russian Federation, and Belarus, the poorest and most heavily affected. Other UN organizations then became more involved. In May of 1991, the WHO headquarters (WHO/HQ) set up the International Project on the Health Effects of the Chernobyl Accident (IPHECA) with > $20 million in funding, primarily from Japan. By that time, the European Regional Office of the WHO (WHO/EURO) had a strong program in place, following its initial response to the accident, to assist its member states other than the USSR in their responses to the accident. In October 1991, WHO/EURO opened an office in Rome with an assignment including the effects of ionizing radiation on health; this office quickly became involved with the affected countries. Over the following year or two, the UN Office for the Coordination of Humanitarian Affairs (OCHA) undertook fundraising and provided humanitarian assistance for the three now very economically disadvantaged countries, as did the UN Educational Scientific and Cultural Organization (UNESCO) (in recognition of the psychosocial consequences), the European Commission (EC), the Red Cross, the Sasakawa Foundation from Japan, the United States, Netherlands, Germany, and several other countries, nongovernmental organizations, and charities. Many of these organizations, the EC, United States, and Japan, among others, also supported research.

Quite early on, attempts were made by the United States, WHO/HQ, and OCHA to coordinate both the humanitarian and research initiatives. One problem was a lack of clarity over the leadership of the newly independent states: the Russian Federation regarded itself as senior to the others, the accident occurred in Ukraine, and Belarus was the most affected country. The United States and WHO/HQ each claimed to have made exclusive agreements with the affected states—IPHECA to the effect that it was to be an umbrella under which all research and humanitarian activities would be coordinated, and the United States to the effect that it had priority where the conduct of research was concerned. OCHA claimed that its mandate overrode other humanitarian-linked agreements. The result was a serious lack of coordination and a fair measure of chaos on both humanitarian and research fronts.

The realization that there was a real radiation-related increase in the rare CTC dominated the research. By 1995, excess relative risks for some areas of Belarus were of the order of 200 (Stsjazhko et al. 1995). This meant that almost every case of CTC was related to the accident and to radiation exposure. Studies were carried out to understand the molecular basis of the carcinogenesis and to look for a marker for radiation etiology that would aid the resolution of claims for compensation from nuclear industry workers and atomic test veterans. The U.S. research was carried out with the knowledge that Congress had ordered a reassessment of the thyroid doses from ^131^I from the Nevada atomic weapons test series. In 1992, when the increase in CTC in Belarus was first reported, that reassessment was complete, although not yet made public. It showed that earlier assessments had significantly underestimated the doses. Before Chernobyl, this information would not have caused great concern in the United States because of the belief that, despite its radioactivity, ^131^I was not carcinogenic. It happened that the same National Cancer Institute (NCI) division was responsible for both the national dosimetric reassessment and the post-Chernobyl research. The former (NCI 1997) was not published until after a newspaper leak in 1997; the latter was a well-designed, long-term cohort study of a population of children with assigned thyroid doses, which was not expected to yield results for several decades. Many epidemiologic studies (mainly ecologic) built a strong circumstantial case for a link between exposure to ^131^I and thyroid cancer, definitively established by a case–control study in 2005 (Cardis et al. 2005a). What the research has not so far yielded is a marker for radiation etiology. Chernobyl-related cancers have so far been predominantly papillary cancers and initially showed a high incidence of *RET* gene rearrangements, also found in spontaneous cancers (Nikiforov et al. 1997). Papillary carcinoma has been increasing in incidence over the last half-century. Although partly due to ascertainment, a contribution of radiation from atomic weapons testing, medical, and dental sources cannot be excluded.

The accident at Chernobyl tested the capacities of the relevant international organizations, and their responses left much to be desired. Initially they were faced with the problem that, although many countries were exposed to radioactive fallout, it was regarded as an internal matter by the country in which it occurred. The next difficulty came with the breakup of that country, resulting in three separate countries containing heavily exposed populations. When outside assistance was eventually welcomed, there were many separate initiatives, and the level of coordination left a great deal to be desired.

The response of the WHO was hampered by internal disagreements. The $20 million used by WHO/HQ to fund the IPHECA program seems to have been largely spent on unproductive pilot projects and on providing training and laboratory and medical diagnostic equipment for the three countries. The largest share went to Russia, despite the fact that it had the least exposure (WHO 1995). A separate initiative was taken by WHO/EURO, which set up the International Thyroid Project. The project suffered from inadequate funding but tried to achieve coordination of patient care and related research on consequences of iodine deficiency in the three affected countries. The WHO/HQ conference, held in November 1995 in Geneva, focused primarily on health issues. It was poorly prepared and attracted a significant number of dubious reports; the proceedings were not published in an accessible form. The lack of coordination between WHO/HQ and WHO/EURO, the political nature of some decisions of WHO/HQ, and the uncertainty over the division of its responsibility with the IAEA all contributed to its problems. In addition, other international organizations regarded the IPHECA program as such a failure that they were reluctant to collaborate with the WHO and would not accept its leadership as envisaged by the umbrella concept.

The IAEA was invited in 1989 to provide an assessment by international experts of the measures taken by the USSR. A team visited some of the affected areas in 1990, and a detailed report (IAEA 1991) assessed the environmental contamination, radiation exposure, and health effects. Cases of thyroid cancer occurring in exposed children in both Belarus and Ukraine in 1990 were reported to the team but were apparently not followed up (IAEA 1991); the general tenor of the report suggests that they were largely discounted because of the belief that ^131^I had a low carcinogenic risk and that the latent period was too short. The report concluded that “there may be a statistically detectable increase in the incidence of thyroid carcinoma in the future.” The attitude of senior IAEA officials in the next few years was antagonistic toward reports of a radiation-related increase in thyroid carcinoma incidence. The mandate of the IAEA enjoins it to promote the peaceful use of nuclear technology, and this, together with its close links to the nuclear industry, would not make evidence of carcinogenic risks following a nuclear accident welcome news. The WHO seems to accept that the IAEA has the dominant role in the investigation of health effects of nuclear accidents, as clearly indicated in a recent report (Peplow 2006). This situation needs to be reassessed to avoid possible conflicts of interest.

The IAEA meeting in Vienna in 1996 provided a major opportunity for policy development for the coming years. The final statement by the conference president, Angela Merkel, then German Minister of the Environment, could have laid the foundations for a properly funded long-term study of all the potential health effects, but the statement, presumably prepared for her by IAEA officials, failed to provide any support or direction for this (IAEA 1996).

The EC was concerned about the consequences of Chernobyl, which took place in Europe and led to fallout across the European Union. It supported work on the incidence, scientific background and appropriate therapy of the thyroid cancers, and on the psychological consequences. The EC also provided extensive support for humanitarian aspects and to remedy the environmental consequences. It maintained close contact with the United States, but after one joint meeting with WHO/EURO in 1992, the EC very strongly discouraged any collaborative studies with the WHO for the next 5 years. Some collaboration was finally established after an independent group of scientists proposed creating a Chernobyl tumor bank to save unique material for future study. The EC provided core funding, and a collaborative project involving the three affected countries, the EC, United States (NCI), WHO/HQ, and Japan was created (Thomas and Williams 2000).

From 1991, UNESCO operated a very effective psychosocial rehabilitation program opening nine rehabilitation centers for adults and children, especially in areas where relocated people were housed. In particular these centers acted to promulgate reliable information about the risks entailed in living in contaminated areas.

In 2001, the United Nations Development Program mounted a needs assessment mission, which identified exposed populations relocated or continuing to live in contaminated regions that “continue to face disproportionate suffering in terms of health, social conditions, and economic opportunity” (UN 2002). The report (UN 2002) described the most vulnerable groups as facing a “progressive downward spiral of living conditions induced by the consequences of the accident” and outlined a 10-year strategy for tackling and reversing this spiral. A key element of that strategy for recovery was to be a body called the International Chernobyl Research Board (ICRB), with a broad assignment including making recommendations for research. As noted above, the theoretical basis for understanding the effects of radiation on health have been in a state of flux since the early 1990s; the earlier concepts (Appendix) are still adhered to because they underpin the present radiation protection framework. Chernobyl has proved fertile ground for views that dissent from those of the establishment, with claims of much greater health impact based on observations or unsubstantiated risk coefficients; mistrust of many of the major international bodies has led to the perverse equation that dismissal by the establishment necessarily testifies to correctness. The ICRB was therefore envisaged as broader and more inclusive than established bodies such as UNSCEAR, the International Committee for Radiation Protection (ICRP), and the IAEA, and as a forum where all views could be debated in a rational way and mistrust lessened. Rather than creating an ICRB, the Chernobyl Forum, lacking the broader representation originally envisaged, was instigated on the initiative of the IAEA to evaluate the health and environmental consequences of the accident. The health section, led by the WHO/HQ, reported recently (WHO 2005a); this highly technical document (WHO 2005a) builds on an earlier review (UNSCEAR 2000). The report was launched as a landmark digest report, with a press release from WHO/HQ headed, “Chernobyl: The True Scale of the Accident” (WHO 2005b). It states, “A total of up to 4000 people could eventually die of radiation exposure from … Chernobyl.” The emphasis is on reassurance, but it is notable that the headline estimate of deaths is less than half the number that can be derived from the body of the report. Neither is it safe to assume that the very low death rate from thyroid cancer to date will apply to future cases, let alone assume that no further deaths from cancer will occur in the present cases. There is no previous experience of an accident such as this, and the long-term risks cannot be predicted with any certainty either in the heavily exposed areas or in the much wider areas with low-level exposures. Certainly there is a clear indication that there is a risk for low dose and low dose rate exposure (Cardis et al. 2005b; Krestinina et al. 2005), but there are also large uncertainties regarding its magnitude. The least that could have been expected from bodies such as the WHO and IAEA would have been support for long-term studies of such a unique event. Without these studies, society will not be able to assess the future risks associated with nuclear accidents, judge what precautions need to be taken, or plan the proper provision for health care.

## What Can We Learn from the Chernobyl Experience?

Chernobyl was the first major accident to a civil nuclear power plant that released huge amounts of radioactive isotopes into the environment. It came as no surprise that there was worldwide public concern, even where doses to the public were tiny (although because of the large population involved, the collective dose was higher than in the immediately affected areas). There have been many smaller incidents involving accidental public exposure to radiation, most notably the Three Mile Island accident (Pennsylvania), arguably as severe as Chernobyl but with secondary containment (not present in the Chernobyl reactor), which largely prevented release of radioactivity to the environment (Weidner et al. 1980). This public concern results to some degree from a lack of understanding of the effects of ionizing radiation, and it might be assumed that the international scientific community would be well equipped to allay at least some of these fears. Although this was attempted, initially with the International Chernobyl Project and later with three conferences around the 10th anniversary, it had not succeeded in 2001 when the UN needs assessment mission visited the affected regions 15 years after the accident.

As stated above, the lack of scientific consensus is at least in part due to the state of flux concerning the understanding of the ways in which radiation affects health, and it is understandable that bodies such as the ICRP are reluctant to change radiation protection standards. The IAEA is bound by a mandate to follow the ICRP dogma. The WHO should be freer to express alternative views. It is regrettable that the WHO played only a minor role in the International Chernobyl Project, which failed to recognize the importance of the CTCs reported to them.

The WHO and IAEA have both made major contributions, but their failures had a number of implications. The delay in the acceptance of the increase in CTC delayed assistance to the affected countries. The 1995/1996 conferences were to a degree mismanaged and missed a major opportunity to create a framework for the future. The major problems with IPHECA contributed greatly to the lack of international coordination and also meant that the International Thyroid Project was never adequately supported. Perhaps the biggest failure resulted from the widespread belief that the IAEA, and in its wake the WHO, wished to disbelieve or minimize the health consequences of Chernobyl, leaving the suspicion that health detriment was being covered up.

With globalization comes the increasing likelihood that accidents, including nuclear accidents, will occur, with impacts crossing national boundaries or presenting challenges beyond the capacity of individual states. Natural disasters such as hurricanes and earthquakes remind us of the high level of coordination and commitment required to respond effectively to such events. One can hardly celebrate the success of the international community in responding to the Chernobyl accident 20 years after the event.

Declining resources of gas and oil, the recognition of global climate change induced by burning of fossil fuels, and the threat of deliberate cessation of energy supply are forcing many countries to rethink the role of nuclear power in energy supply. As a salutary example of nuclear technology failure, the Chernobyl accident should give pause for thought. Clearly, rational decisions on future energy policy need to be made in light of the risks that alternative strategies incur. It is important to recognize that the accident happened in a reactor lacking secondary containment and that adequate precautions to protect public health were not always taken after the accident.

Action is needed to ensure adequate understanding of the health problems following Chernobyl; this requires creating and funding a structure to allow studies to continue for the lifetime of those exposed to the accident. The creation of the Atomic Bomb Commission (now renamed the Radiation Effects Research Foundation) was critical to understanding the health consequences of atomic bomb exposure. A similar organization could still provide an appropriate framework for Chernobyl studies. The rivalry and lack of coordination between many organizations, UN agencies, countries and others that followed Chernobyl must be avoided in future international-scale accidents. The UN should initiate an independent review of the response to Chernobyl, including the actions and assignments of the UN agencies involved. It should also advise on the organization necessary for future accidents. The Radiation Effects Research Foundation provides a model of international cooperation with independent scientists involved. Chernobyl is undoubtedly a more complicated situation, but the principles of cooperation rather than rivalry and independence remain essential.

We do not believe that the Chernobyl accident should necessarily be regarded as an insurmountable obstacle to future nuclear power development, although any new reactors must have secondary containment. We have stressed the uncertainties involved in predicting the long-term consequences of Chernobyl and believe this approach to be far preferable to either downplaying or exaggerating the risks. We do believe that the responses of the major international organizations to the Chernobyl accident were inadequate and show the need for a review by the United Nations of their assignments and coordination and for the development of a new strategy for dealing with future disasters. Above all, we believe that it would be a dereliction of duty by the world community if it did not ensure continuing study of the consequences of a tragic accident that we hope will never recur.

## Appendix

Ionizing radiation is capable of bringing about chemical modification of genomic DNA, that is, mutating the base sequence of genes; it is this feature that has traditionally been assumed to be the basis of the health-damaging effects of radiation. Where the appearance of health detriment is delayed by months to years, as in the case of cancer, it was assumed that specific genes became mutated in ways that either promoted inappropriate growth of cells in the affected tissue or failed to suppress such growth. Thus, the extent, in terms of dose, to which radiation was able to initiate cancer, for example, was related to the chances of damaging a specific gene. Radiation-induced events are randomly distributed in the exposed cells and can be viewed as bullets fired from a scattergun: the smaller the target gene sequence that has to be damaged, the larger the dose (number of bullets) required. Thus, effect and dose were intimately related through the distribution of radiation damage in the DNA of the cells of the irradiated tissue.

In 1991, a new radiation-induced phenomenon was reported from laboratory experiments, namely, genomic instability (Morgan et al 1996). This phenomenon comprises the delayed induction of changes by radiation (e.g., mini-satellite DNA mutations, chromosomal damage, sequence mutations, micronucleus formation), which cannot be due directly to damage to specific DNA sequences because the radiation doses at which they are instigated are too small. Subsequently, a second effect—termed the bystander effect—occurs when cells not irradiated themselves, but in the vicinity of cells that are irradiated, also exhibit these effects. These two effects cannot be subsumed into a theoretical framework that has as its basis the distribution and location of specific damage caused by ionizing events.

Many of the effects characteristic of instability and the bystander effect are present in malignant cells. This has led to the proposal that genomic instability may be a precursor to malignancy (Little 2000).

It has been proposed that genomic instability is a generic response of the genome to damage to its genomic DNA (Baverstock 2000) and that it is intimately connected with the processes that endow the genome with stability. Whatever the fundamental basis, genomic instability challenges the existing dogma (Baverstock and Belyakov 2005), particularly with respect to what happens at low doses and low dose rates, a domain that is difficult to explore with epidemiologic techniques.
